# High-throughput multiplex qPCRs for the surveillance of zoonotic species of canine hookworms

**DOI:** 10.1371/journal.pntd.0008392

**Published:** 2020-06-15

**Authors:** Luca Massetti, Vito Colella, Patsy A. Zendejas, Dinh Ng-Nguyen, Lana Harriott, Lara Marwedel, Anke Wiethoelter, Rebecca J. Traub

**Affiliations:** 1 Faculty of Veterinary and Agricultural Sciences, University of Melbourne, Parkville, VIC, Australia; 2 Faculty of Animal Sciences and Veterinary Medicine, Tay Nguyen University, Dak Lak, Vietnam; 3 Pest Animal Research Centre, Biosecurity Queensland, Department of Agriculture and Fisheries, Toowoomba, QLD, Australia; 4 Boehringer Ingelheim Animal Health Australia, North Ryde, New South Wales, Australia; University of Cambridge, UNITED KINGDOM

## Abstract

The canine hookworms *Ancylostoma braziliense*, *Ancylostoma ceylanicum*, *Ancylostoma caninum* and *Uncinaria stenocephala* are not only capable of producing morbidity and mortality in dogs but are also neglected tropical zoonoses. Each hookworm species differs considerably in its geographical distribution, life cycle, biology, pathogenic impacts on both canine and human hosts, zoonotic potential, and response to treatment with anthelminthics. Here we describe the development and validation of two Taq-Man based multiplex PCR assays capable of detecting and differentiating all four canine hookworm species in faeces of naturally infected dogs. The analytical sensitivity of both assays was assessed using 10-fold serial dilutions of synthetic gene block fragments containing individual sequence targets of each hookworm species. The sensitivity of the assays and ability to detect mixed species infections were compared to a conventional PCR-Restriction Fragment Length Polymorphism based-approach when applied to laboratory and field samples from endemic areas.

The qPCRs detected at least one species of hookworms in 82.4% of PCR-RFLP-negative but microscopy-positive samples. The qPCRs detected an additional 68% mixed infections with different species of canine hookworms, and additional single species infection with *A*. *caninum* (47%), *U*. *stenocephala* (33%) and *A*. *ceylanicum* (0.02%) that were missed by PCR-RFLP. These multiplex qPCR assays will assist field based epidemiological surveillance studies towards an accurate and sensitive monitoring of canine hookworm infections in dogs, to inform their species-specific zoonotic risks to populations living in endemic areas, globally.

## Author summary

There are four species of blood-sucking hookworms that infect dogs, namely *Ancylostoma braziliense*, *Ancylostoma ceylanicum*, *Ancylostoma caninum* and *Uncinaria stenocephala*. Each hookworm species differs in its geographical distribution, impact on canine health and more importantly its ability to infect and produce disease in humans. For example, dogs are the primary reservoir for *A*. *ceylanicum*, the second most common hookworm species infecting humans in the Asia Pacific. *Ancylostoma braziliense* is more geographically confined and responsible for chronic skin eruptions in humans. Accurate diagnosis of hookworm species in dogs is therefore essential for understanding the relative risk each species pose to human populations living in close association with dogs. As microscopic methods are unable to distinguish between species of hookworms in stool, highly efficient real time PCR assays capable of detecting and differentiating all four canine hookworm species in canine faeces were developed. These new assays are superior to previously published gel-based PCR assay displaying higher throughput, and ability to correctly identify single and mixed hookworm species infections with higher accuracy.

## Introduction

The canine hookworms *Ancylostoma braziliense*, *Ancylostoma ceylanicum*, *Ancylostoma caninum* and *Uncinaria stenocephala* are widely distributed soil-transmitted helminths causing morbidity in dogs and are agents of zoonoses [1–8]. Each hookworm species differs considerably in its geographical distribution, life cycle, biology, pathogenic impact on both canine and human hosts, zoonotic potential and response to anthelminthic treatment [1,9–15]. Dogs and humans become infected percutaneously or orally via the ingestion of the ensheathed larvae in soil or via contaminated food or water [15]. For *A*. *caninum*, *A*. *braziliense* and *U*. *stenocephala* transmission by dogs preying on paratenic hosts is also possible [6–8,16]. Trans-mammary transmission in dogs also occurs for *A*. *caninum* [15,17,18]. Infection, especially with *A*. *caninum*, is a common cause of haemorrhagic diarrhoea and death in pups and chronic iron deficiency anaemia in adult animals [18,19]. All canine hookworm species cause cutaneous larva migrans (CLM) [20]. In human patients, *A*. *braziliense* is by far the most frequently implicated, as it is the only species capable of causing classical ‘creeping eruptions’, a prolonged highly pruritic and serpiginous eruption within the dermis that may persist for over 100 days, if untreated [9–11]. In humans, *A*. *caninum* is a well-recognized agent of eosinophilic enteritis and aphthous ileitis. Although most infections are asymptomatic, a single immature adult worm residing in the small intestine is capable of eliciting abdominal pain, intestinal bleeding, diarrhoea and weigh loss [13,21,22]. Recent findings of *A*. *caninum* eggs in the faeces of human patients highlights its potential to complete its life cycle in humans [23].

*A*. *ceylanicum* on the other hand, is the only canine (and feline) hookworm species known to produce patent, long-lived infection within the intestinal tract of humans both in both natural and experimental infections [24–30]. In the Asia Pacific *A*. *ceylanicum* is the second most common hookworm infecting humans after *Necator americanus* and is responsible for between 5.5–51.6% of human infections in Laos, Malaysia, Thailand, Cambodia and the Solomon Islands [30–35]. *Ancylostoma* spp. are mostly found in subtropical and tropical regions and may co-exist as sympatric species and/or be featured by specific ecological requirements with implications for their distribution. For instance, *A*. *caninum* is endemic in wet and dry regions of the tropics and subtropics globally, while *A*. *ceylanicum* thrives in the wet tropics and subtropics of Africa, Asia and Oceania. *Ancylostoma braziliense* appears limited in distribution to a narrow latitudinal areas spanning approximately 5°N to 10°S of Brazil, Kenya, Tanzania, northern Australia, Indonesia and Malaysia [1–5]. Conversely, *U*. *stenocephala*, inhabits predominantly temperate and colder climate such as those of Europe, Canada, southern Australia and the United States of America [14,36].

In semi-domesticated dogs residing in urban and rural areas of Asia, Africa and South America, between 40–100% of dogs may be infected with hookworms contributing to a high environmental dispersal of potentially zoonotic infectious larvae [21, 29–32]. The high proportion of *Ancylostoma*-infected dogs in these tropical climates is exacerbated by the co-existence of several risk factors that allow the parasite to thrive in dogs and significantly increase their risk of infecting humans i.e. free ranging community dogs, a lack of veterinary care, irregular deworming, roaming behaviour and poor environmental hygiene [35,37–39].

Diagnosis of canine hookworm infections has traditionally relied on the identification of eggs isolated in faecal floatation and species-based identification by examining adult worms following necropsies or anthelmintic treatment [14,40]. For research purposes, these traditional techniques were replaced with molecular-based diagnostic assays nearly two decades ago, including PCR-Restriction Fragment Length Polymorphism (PCR-RFLP) [41,42] targeting the internal transcribed spacer (ITS)-2 region as genetic marker for the detection and discrimination of hookworms from canine faeces. The PCR-RFLP, however, is relatively labour-intensive limiting large-scale epidemiological studies. Here we present novel Taq-Man based multiplex qPCRs to screen for canine hookworm species in mixed natural infections from canine faeces. Diagnostic parameters of the multiplex qPCR assays were validated and compared to the PCR-RFLP by using genomic DNA sourced from previously published epidemiological studies for canine hookworms conducted in Vietnam [43] and Southeast Queensland, Australia [44]

## Materials and methods

### Parasite material

A total of 191 faecal samples previously demonstrated positive for *A*. *ceylanicum*, *A*. *caninum* and *U*. *stenocephala* by PCR-RFLP were selected [42]. Of these 191 samples, a subset of 111 samples were also subjected to sodium chloride floatation and identified positive for hookworm eggs. Faecal samples were sourced from an existing collection donated by Dr. Dinh Ng-Nguyen from a previous investigation in 2014 in Vietnam [43] and from Dr. Lara Harriot from an investigation performed from 2012 to 2015 in Queensland (Australia) [44]. Of these 191 faecal samples 54 were confirmed positive for *A*. *caninum*, 60 *A*. *ceylanicum* and 4 *U*. *stenocephala* by PCR-RFLP [42]. Twelve samples harboured mixed infections with two or more species of hookworm. Hookworm-negative faeces were spiked with DNA of *A*. *braziliense*. This negative faecal sample was collected from a fully-dewormed pet dog residing in Melbourne, Victoria, and demonstrated free of hookworm eggs using sodium nitrate faecal floatation (SG 1.20). The hookworm-free faecal sample was divided into equal aliquots and spiked with serial dilution of genomic *A*. *braziliense* DNA belonging to an existing collection of hookworm genomic DNA of Prof. Rebecca Traub (University of Melbourne). DNA concentrations were quantified using a Qubit 4 Fluorometer (Life Technologies, Thermo Fisher Scientific Inc.) as per manufacturer’s instructions. Ten-fold serial dilutions of 0.219 ng/μl genomic DNA were prepared and spiked into seven tubes containing 150 mg of faeces each and 150 mg faeces with nuclease free water was used as negative control.

DNA was extracted from faeces using the ISOLATE Fecal DNA Kit (Bioline Sydney, Australia) according to the manufacturer’s instructions. Final DNA elution was made in 100 μl of elution buffer.

### Multiplex qPCRs

The multiplex qPCR assays were designed to detect the ITS-1 rRNA region of *A*. *braziliense*, *A*. *ceylanicum*, *A*. *caninum* and *U*. *stenocephala*. Reference nucleotide sequences for the ITS-1 region of *A*. *caninum* (Reference GenBank accession number JQ812694; KP844730; DQ438071), *A*. *ceylanicum* (Reference GenBank accession number DQ780009; DQ831518), *U*. *stenocephala* (Reference GenBank accession number HQ262054; AF194145) and *A*. *braziliense* (Reference GenBank accession numbers JQ812692; DQ359149; DQ438056) were obtained from NCBI and aligned with GenBank sequences sourced from the available range of geographical isolates for each hookworm species, as well as other closely related canine helminths using Clustal Omega (https://www.ebi.ac.uk/Tools/msa/clustalo/) (S1 Text). Primers and probes targeting highly conserved region of the ITS-1 were designed manually and analysed for suitability within each multiplex assay using Oligo Analyzer (Integrated DNA technologies, https://sg.idtdna.com/calc/analyzer) and their genus or species-based specificity assessed using NCBI Blast (http://www.ncbi.nlm.nih.gov/blast). The primers were manufactured by Integrated DNA Technologies, USA. The probes were designed and manufactured using LNA (locked nucleic acid) technology (Integrated DNA Technologies, USA) to increase probe specificity. A four-channel Magnetic Induction Cycler (BioMolecular Systems, Sydney, Australia) was used for the amplification, detection, and data analysis (micPCR software).

Two qPCR multiplex assays were developed. The first reaction used a common primer pair designed (AcanceyF and AcanceyR) to amplify a 103 bp region of *A*. *caninum* and *A*. *ceylanicum* ITS-1 rRNA. The second qPCR used a common primer pair UncbrazF and UncbrazR to amplify a 119 bp and 118bp region of the ITS-1 rRNA of *A*. *brazilienze* and *U*. *stenocephala*, respectively. Equine herpes virus (EHV4) primers (EHVF and EHVR), probe (EHV probe) and genomic DNA were used as reaction internal control, and mammalian primers (Mam F and Mam R) and probe (MAM PROBE) designed to target a 92 bp region of the 16S mitochondrial gene of mammals was used as a DNA extraction control for both qPCR assays.

Optimisation of each singleplex and multiplex assays were carried out using synthetic double stranded DNA fragments (gBlocks Gene Fragments, IDT Technologies, Skokie, Illinois, USA) containing individual sequence targets of each hookworm species. Progressive concentrations of primer pairs and probes were tested to select the concentrations that guaranteed the highest efficiency and the greatest sensitivity.

The qPCR assays were conducted in 20 μL reactions containing 10 μL of GoTaq Probe qPCR Mastermix (Promega, Madison, WI), 1 μL of known quantity of EHV4 gDNA, and 2 μL of template DNA. Nuclease free-water was added to reach the final reaction volume. The cycling threshold was set at 0.2 units for *A*. *caninum* and *A*. *ceylanicum* and 0.1 unit for *A*. *braziliense* and *U*. *stenocephala*. The fluorescent threshold was set to 5% for all targets. The cycling conditions were identical for both reactions: 2 min at 95°C, followed by 40 cycles of 15 sec at 95°C and 1 min at 60°C. gBlocks Gene Fragments were used as positive controls and nuclease-free water was used as negative control in all runs. The efficiency and the intra-assay reproducibility of multiplex reactions were directly compared to each singleplex reaction by plotting Ct versus 10-fold serial dilutions of target gBlock Gene Fragments of known DNA concentration. The correlation coefficients (R^2^) of the standard curves were produced automatically by micPCR software (BioMolecular Systems, Sydney, Australia).

### Sensitivity and specificity of the qPCRs

The analytical sensitivity (i.e. the ability of an essay to detect low concentrations of a given target substance or organism in a biological sample [45]) of the qPCRs was assessed using 10-fold serial dilutions of target gBlock Gene Fragments of known DNA concentration (i.e., 1.8 ng/ μl for *A*. *caninum*, 7,17 ng/μl for *A*. *ceylanicum*, 13.2 ng/μl for *A*. *braziliense* and 7.22 ng/ μl for *U*. braziliense). In addition, the analytical sensitivity of the qPCRs and PCR-RFLP was assessed and compared using 10-fold dilutions of known concentration of genomic DNA (i.e. 1.14 ng/ μl for *A*. *ceylanicum*, 1.65 ng/ μl for *A*. *caninum*, 2.94 ng/ μl for *A*. *braziliense* and 3.94 for *U*. *stenocephala*) sourced from an existing collection of hookworm genomic DNA of Prof. Rebecca Traub (University of Melbourne).

To investigate the analytical specificity of the assay (i.e. the ability of an assay to exclusively identify a particular target substance or organism rather than similar but different substances or organisms [45]) genomic DNA of the most common parasites infecting dogs, namely *Echinococcus granulosus* sensu lato, *Dipylidium caninum*, *Taenia* spp., *Spirometra erinacei*, *Strongyloides stercoralis*, *Giardia duodenalis*, *Trichuris vulpis*, *Toxocara* spp., and *Neospora caninum* were used. Additionally, we tested the newly developed qPCRs against the common human hookworm *Necator americanus*.

The diagnostic sensitivity (i.e. the ability of an assay to correctly classify an individual as positive if the individual is truly positive [45]) was assessed on a subset of 111 microscopy positive samples previously screened for hookworm eggs using sodium nitrate solution with S.G. 1.20 [43]. The 111 microscopy positive samples were subjected to the multiplex qPCRs and to the PCR-RFLP. As microscopy can be considered a gold standard to establish diagnosis (i.e. microscopy positive samples are truly positive for hookworm infection), the overall diagnostic sensitivity of the qPCRs was calculated as the number of hookworms correctly identified by the qPCRs (true positives) divided by the total number of microscopy positive samples (true positives and false negatives).

Assessment of the overall diagnostic specificity (i.e. the ability of an assay to correctly classify an individual as negative if the individual is truly negative) of the qPCRs was not possible due to truly negative samples being unavailable. Given that microscopy negative samples cannot be considered as truly negative for hookworm infection [46], microscopy cannot be considered a gold standard to rule out infection. Using an imperfect gold standard (i.e. assays with less than 100% diagnostic sensitivity and specificity) would lead to biased results [47–51].

Comparison of the PCR-RFLP and qPCRs was therefore performed relying on Kappa statistics [52] using Excel 2016 (Microsoft Corp., Redmond, WA). Agreement between both assays was considered poor if the coefficient (*k*) <0.00, slight if 0.00*≤ k* ≤0.20, fair if 0.21≤ *k* ≤0.40; moderate if 0.41≤ *k* ≤0.60, substantial if 0.61≤ *k* ≤0.80 and almost perfect if *k* >0.80. The 95% confidence intervals (95% CI) were calculated using the Wald method.

## Results

### Canine hookworm Multiplex qPCR optimization

**Fig 1:**
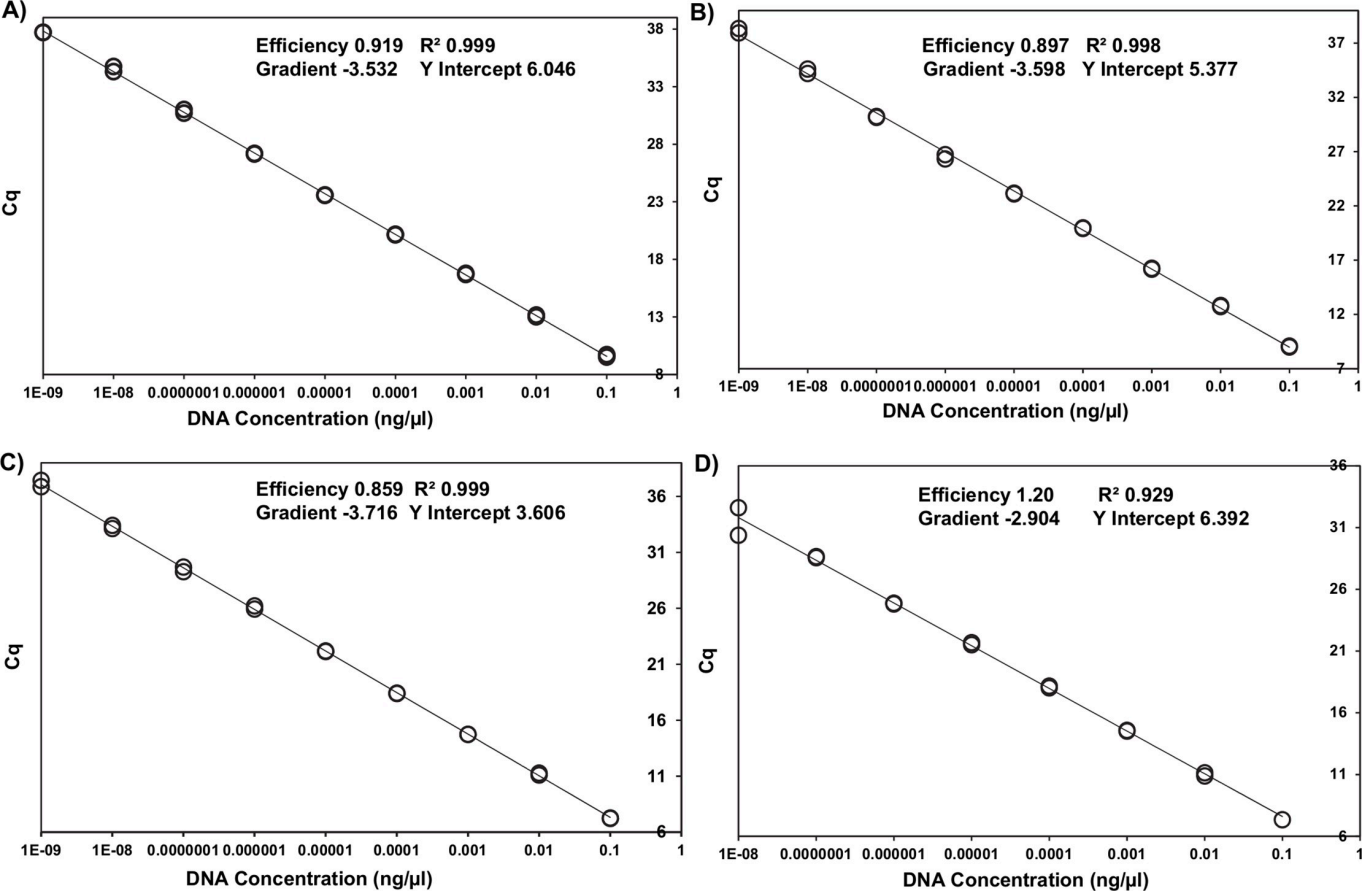
Standard curves generated from 10-fold serial dilutions of target gBlock Gene Fragments of *U*. *stenocephala* (A), *A*. *braziliense* (B), *A*. *ceylanicum* (C), and *A*. *caninum* (D).

The optimized primer and probe concentrations are listed in Table 1. qPCRs efficiencies ranged from 86% to 120% with an R^2^ from 0.929 to 0.999 and Slope from -2.904 to -3.716 (Fig 1).

**Table 1:** Oligonucleotide primers and probes for qPCR assays for the detection of *A*. *caninum*, *A*. *ceylanicum*, *A*. *braziliense*, and *U*. *stenocephala*.

Target	Primers and probes	Sequence (5’–3’)	Target	Size (bp)	Conc. nm	Source
Hookworms	ACancey F	GGG AAG GTT GGG AGT ATC G	ITS-1	103	300	This study
	AcanceyR	CGA ACT TCG CAC AGC AAT C			300	This study
*A*. *caninum*	Acan probe	5HEX/AG+T+CGT+T+A+C+TGG/3IABkFQ			100	This study
*A*. *ceylanicum*	AceyDOGprobe	5Cy5/CCGTTC+CTGGGTGGC /3lAbRQSp			100	(Hii et al., 2018)
Hookworms	UncbrazF	GAG CTT TAG ACT TGA TGA GCA TTG	ITS-1		700	This study
	UncbrazR	GCA GAT CAT TAA GGT TTC CTG AC			700	This study
*A*. *braziliense*	AbraProbe	56FAM/TGA GCG CTA /ZEN/GGC TAA CGC CT/3IABkFQ/-3'		119	200	This study
*U*. *stenocephala*	UncProbe	5HEX/CAT TAG GCG /ZEN/GCA ACG TCT GGT G/3IABkFQ		118	200	This study
Canine DNA	MAM F	CGACCTCGATGTTGGATCAG	16S MtrRNA gene	92	100	(Hii et al. 2018)
	MAM R	GAACTCAGATCACGTAGGACTTT			100	(Hii et al. 2018)
	MAMPROBE	FAM/CCTAATGGT/ ZEN/ GCAGCAGCTATTAA/ LABKFQ			200	This study
Equine Herpes Virus	EHV FWD	GATGACACTAGCGACTTCGA	gB gene	81	80	(Bialasiewicz et al., 2009)
	EHV-REV	TTTCGCGTGCCTCCTCCAG			80	(Bialasiewicz et al., 2009)
	EHV PROBE	ROX/TTTCGCGTGCCTCCTCCAG/3IAbRQSp			200	(Bialasiewicz et al., 2009)

+ Locked Nucleid Acid (LNA) basis

**Fig 2:**
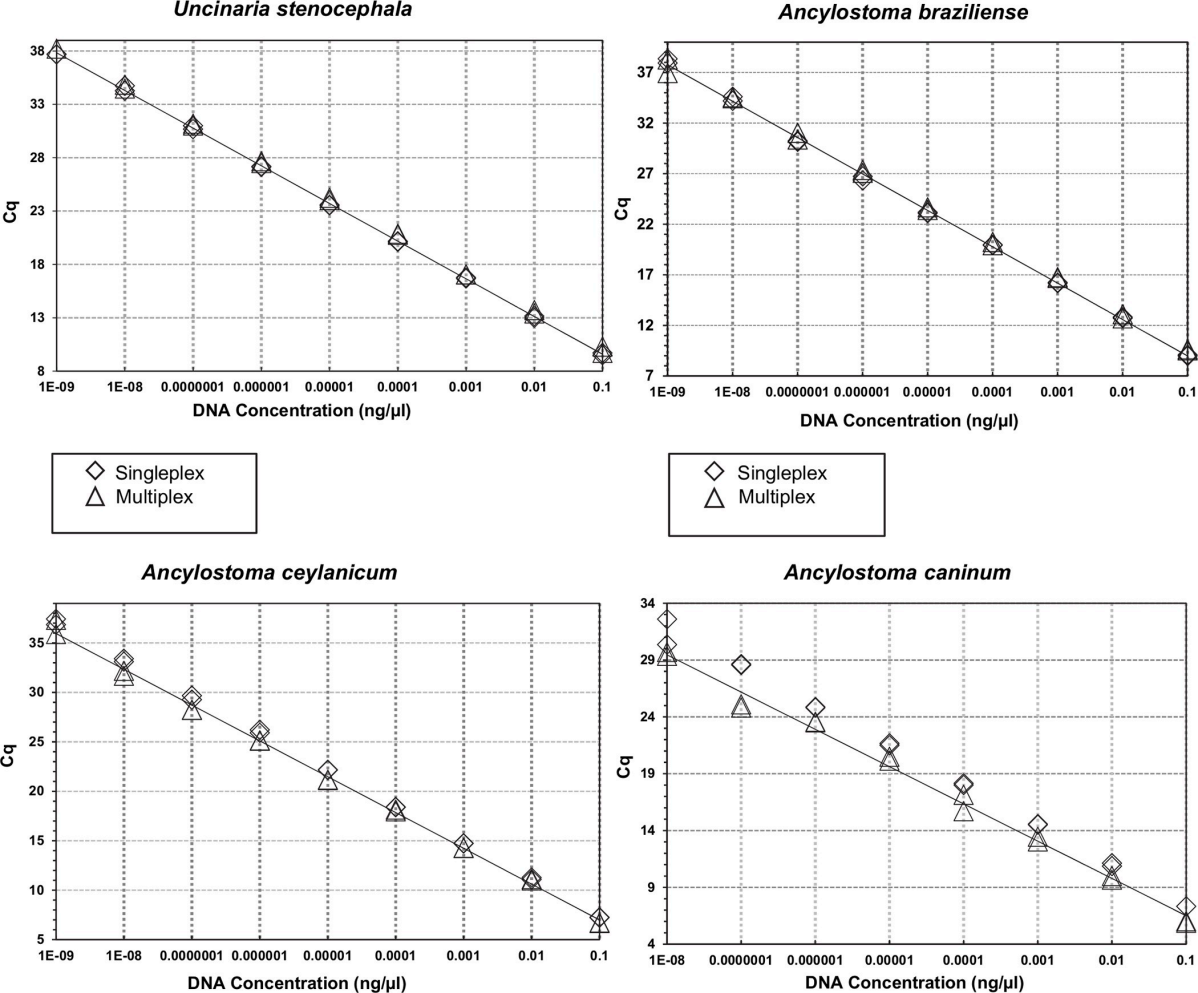
Singleplex and multiplex qPCRs efficiencies. Optimisation and comparison of the sensitivity and efficiency of each singleplex and multiplex qPCRs using gBlock Gene Fragments controls for *U*. *stenocephala*, *A*. *braziliense*, *A*. *ceylanicum*, *A*. *caninum*.

There were no significant differences between the efficiency, analytical sensitivity and intra-reaction reproducibility between singleplex and multiplex assays (Fig 2). No cross reactions between targeted hookworm species were observed for either singleplex or multiplex assays using gBlock Gene Fragments or genomic DNA controls.

### Sensitivity and specificity of the qPCRs

The analytical sensitivity of the assay was 0.0146 ng genomic DNA of *A*. *braziliense* per mg of spiked faeces. Mean Cq values associated corresponding to minimal detection limits were 37.66 (S.D.± 0.05) for *U*. *stenocephala*, 38.27 (S.D.± 0.06) for *A*. *braziliense*, 36.89 (S.D.± 0.368) for *A*. *ceylanicum* and 37.62 (S.D. 0.30) for *A*. *caninum* corresponding to 1.4 x 10^−3^ fg of target gBlock Gene DNA for *A*. *ceylanicum*, 2.6 x 10^−3^ fg of target gBlock Gene DNA for *A*. *braziliense*, 1.4 x 10^−3^ fg for *U*. *stenocephala* and 3.8 x 10^−4^ fg for *A*. *caninum*.

The limit of detection of the qPCRs and PCR-RFLP were respectively 0.00028 ng and 0.0028 ng for *A*. *ceylanicum*, 0.00033 ng and 0.033 ng for *A*. *caninum*, 0.00058 ng and 0.0058 ng for *A*. *braziliense*, and 0.0078 ng and 0.78 ng for *U*. *stenocephala*.

The multiplex qPCRs displayed a diagnostic sensitivity using microscopy as the gold standard of 97.3% (108/111; 95% CI 92.01–99.42). The diagnostic sensitivity of the PCR-RFLP against microscopy was 84.7% (94/111; 95% CI 76.74–90.31).

Neither multiplex qPCR assays cross reacted with genomic DNA of the tested parasites. No cross reactions with other target hookworm species was observed, thereby showing high analytical specificity.

### Performance of the multiplex qPCRs for species of canine hookworm

**Fig 3:**
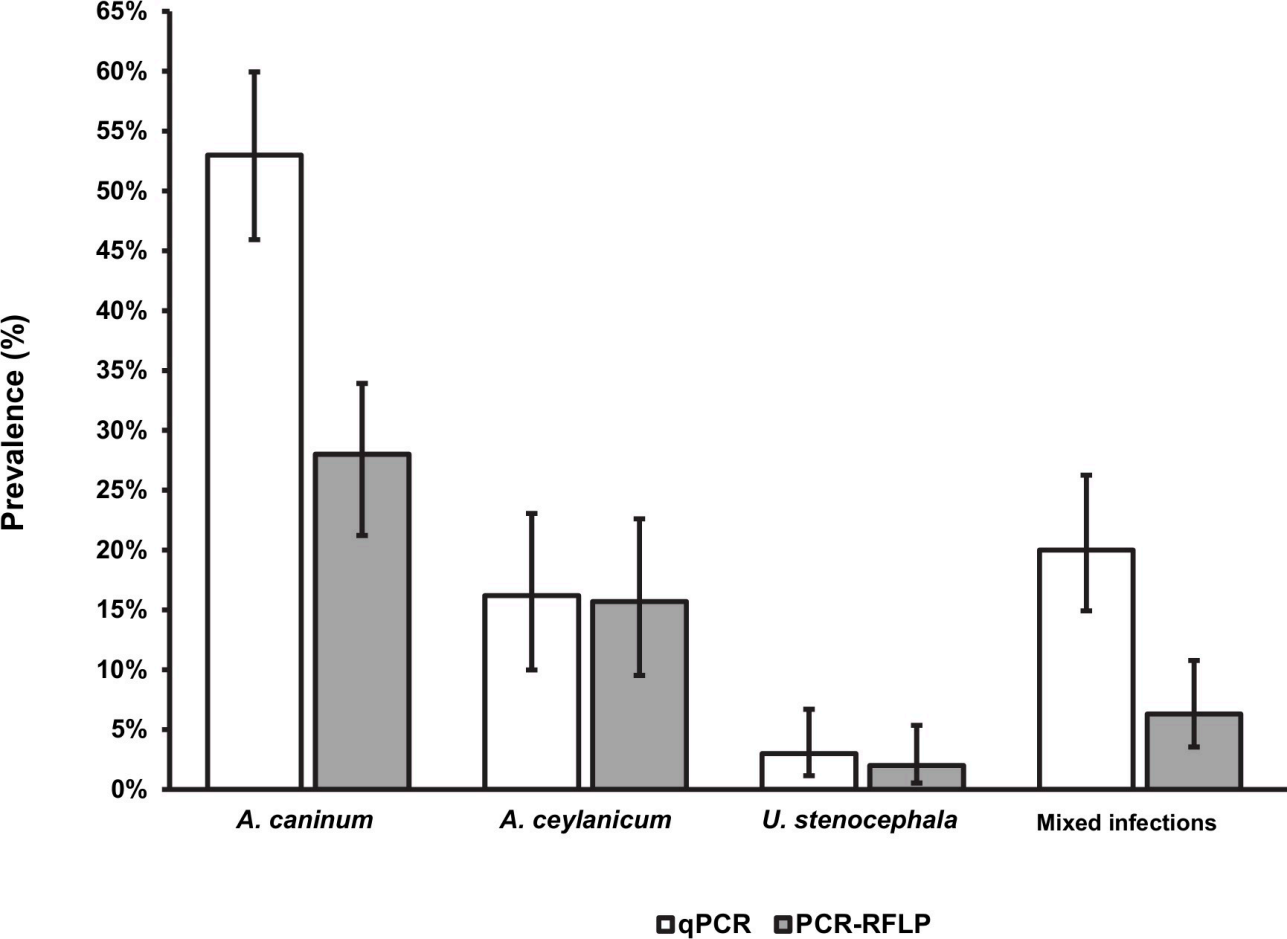
Comparison between multiplex PCR-RFLP and multiplex qPCRs. Conventional PCR results in grey, qPCRs results in white.

Performance of the canine hookworm multiplex qPCRs were determined using 191 DNA samples sourced from previous canine hookworm surveys in Vietnam and Queensland. Field samples, positive controls, EHV4 and mammalian DNA amplified within the expected Cq values. The detection of the signal derived from the amplification of mammalian DNA in all the samples confirmed the success of the extraction step. The multiplex qPCRs showed greater diagnostic sensitivity for the detection of hookworms in comparison to the PCR-RFLP (97% and 84%, respectively). The results for the multiplex quantitative qPCR and the PCR-RFLP are summarized in Table 2 and compared in Fig 3. Kappa statistics reported in Table 2 showed only fair agreement between the qPCRs and the PCR-RFLP for mixed infections (κ = 0.25, 95% CI 0.02–0.48) and *A*. *caninum* (κ = 0.43, 95% CI 0.31–0.56). Substantial agreement was shown for *A*. *ceylanicum* (κ = 0.79, 95% CI 0.7–0.89) and *U*. *stenocephala* (κ = 0.79, 95% CI 0.51–1).

**Table 2:** Multiplex quantitative PCR and PCR-RFLP agreement statistics.

		PCR-RFLP			
	qPCRs	POS	NEG	Total agreement %	Kappa (95% CI)
***A*. *caninum***	POS	50	52	70%	0.43 (0.31, 0.56)
	NEG	4	85		
***A*. *ceylanicum***	POS	52	9	91%	0.79 (0.7, 0.89)
	NEG	8	122		
***U*. *stenocephala***	POS	4	2	99%	0.79 (0.51, 1)
	NEG	0	185		
**Mixed infections**	POS	8	30	82%	0.25 (0.02, 0.48)
	NEG	4	149		

POS = positive; NEG = negative; 95% CI = 95% confidence interval

K agreement level: k) <0.00 poor; 0.00≤ k ≤0.20, slight; 0.21≤ k ≤0.40, fair; 0.41≤ k ≤0.60, moderate; 0.61≤ k ≤0.80; substantial; k >0.80 almost perfect.

## Discussion

Here we propose and validate two Taq-Man probe-based multiplex qPCRs for the detection of zoonotic species of canine hookworms. These diagnostic tools will assist large epidemiological field studies toward a timely surveillance and monitoring of canine hookworm infections in dogs and provide information on species-specific zoonotic risks to populations living in endemic areas, globally.

While several qPCR assays have been developed for high-throughput screening of human hookworm species [46,53–55], there is a considerable lack of diagnostic tools to understand species distribution of canine hookworms. During the last decade, the development of qPCRs targeting human hookworms has aided monitoring of the distribution, efficacy and impact of mass deworming programs targeting individual genera or species of both anthroponotic and zoonotic hookworms [46,53,54]. The zoonotic species of canine hookworms are emerging pathogens with *A*. *ceylanicum* alone estimated to infect up to 73 million people in the Asia Pacific [30–35]. Similarly, the development and field application of a multiplex qPCR assay for canine hookworm species can be applied to assess their distribution, inform zoonotic risk, facilitate field-based anthelmintic efficacy trials and monitor the impacts of One Health intervention strategies for their control.

The qPCRs herein described demonstrated higher diagnostic sensitivity (97%) in comparison to previously developed PCR-RFLP (84%) by Ng-Nguyen et al. (2015). For instance, our qPCRs successfully detected at least one species of hookworms in the 82.4% of PCR-RFLP-negative but microscopy-positive samples. The absolute sensitivity of the qPCRs was 10-fold more sensitive than the PCR-RFLP for *A*. *ceylaniucm* and *A*. *braziliense* and 100-fold more sensitive for *A*. *caninum* and *U*. *stenocephala*.

One of the main advantages of our qPCRs was the capability of detecting additional mixed hookworm species infections (68%) compared to PCR-RFLP. In addition, the qPCRs detected an additional 48 *A*. *caninum* single infections (47%), two *U*. *stenocephala* (33%) and one *A*. *ceylanicum* (0.02%) missed by PCR-RFLP. A small number of samples were qPCR-negative and PCR-RFLP positive however, it is likely that these samples were PCR-RFLP false-positives associated with the subjective and potentially inaccurate interpretation of gel electrophoresis results. These assays have proven specific as it detected all the four canine hookworms tested without cross reacting when tested against a range of 10 different intestinal parasites of dogs and the human hookworm *N*. *americanus*.

The primary disadvantage of the qPCR is its inability to differentiate *A*. *caninum* from *A*. *duodenale* DNA, given the high degree of nucleotide identity between DNA sequences of these two closely related hookworm species. The ability to differentiate canine from human hookworm species becomes apparent in resource-poor communities that practice outdoor defaecation, as dogs are known to be coprophagic [56]. Moreover, *A*. *duodenale* has been demonstrated to produce self-limiting infections in experimentally infected puppies in a non-controlled study [57], a phenomenon that is likely to be rare in natural *A*. *caninum*-endemic settings as the transmammary route of infection in pups is likely to provide cross-protective immunity against its closely related human-hookworm counterpart. Despite this limitation, the likelihood of false positive *A*. *caninum* infection in dogs as a result of the coprophagy or patent infections with *A*. *duodenale* appears negligible. DNA of *Ancylostoma duodenale* was detected in a single canine faecal sample in Turkana, Kenya at an overall prevalence of <0.062%, even in the presence of widespread canine coprophagic behaviour [58].

Kappa statistics showed only fair agreement (0.41 ≤|κ|≤ 0.6) between the qPCRs and the PCR-RFLP for mixed and single *A*. *caninum* infections. The primary reason for this was the qPCRs assays’ increased sensitivity for the detection of *A*. *caninum* and mixed hookworm-species infections. Good agreement (0.61 ≤|κ|≤ 0.80) was instead shown for *A*. *ceylanicum* and *U*. *stenocephala*.

The primary limitation of the study is that only microscopy positive samples were subjected to the PCR-RFLP assays and therefore diagnostic specificity was not assessed. Further studies testing the relationship between Cq values/copy numbers to egg numbers are needed before these qPCRs can be used as quantitative tools to assess infection intensity levels. The deployment of more sensitive techniques such as the qPCR assays herein validated, acquires pivotal importance especially in areas where preventive chemotherapy has been employed for many years and where conventional techniques may fail to detect light-intensity infections (i.e. shedding low number of eggs) [59,60]. The advantages of these high-throughput qPCRs over the previously published assays traditionally used for the diagnosis of canine hookworms is not only a greater sensitivity and specificity but also its reduced processing time [46]. Even though qPCR may be slightly more expensive than PCR-RFLP, this disadvantage is overcome by significantly reduced labour costs associated with shortened qPCR set-up times and real-time analysis of results.

These newly developed qPCRs will facilitate large-scale detection and proper identification of canine hookworm species, globally, by researcher dealing with the assessment of STHs in endemic areas. Further, this tool will aid in promptly diagnosing and treating affected animals by veterinary clinicians whom can refer cases to diagnostic laboratories. Further, faecal samples can be easily stored and transported at room temperatures to laboratories with qPCR capabilities without the need of trained technicians or special equipment, making it an ideal tool for large survey especially when sampling animals living in remote areas. These new tools provide an alternative to the labour-intensive microscopic examination of faecal samples and to low-throughput gel-based PCR-RFLP for the diagnosis of canine hookworm infections.

These qPCRs will enable a comprehensive investigation of the epidemiology of canine hookworm infections in dogs and their risk posed to humans especially in areas where *A*. *ceylanicum*, *A*. *caninum* and *A*. *braziliense* are sympatric. Its greater accuracy in comparison with PRC-RFLP may be explained owing to outputs being based on an objective automated quantitation of fluorescent signals instead of a visual-based size discrimination of the bands in an electrophoresis gel.

Distinguishing between the four species of canine hookworm is also important to inform One Health based chemotherapeutic intervention strategies in dogs, as species-specific differences in anthelmintic efficacy have been reported [1,9–15]. For example, certain geographical isolates of *A*. *caninum*, the well-known cause of eosinophilic enteritis in humans, have demonstrated high-level of pyrantel resistance [61]. Poor efficacy of milbemycin oxime against *U*. *stenocephala* has also been demonstrated [62].

Furthermore, although hookworm eggs are morphologically indistinguishable, the zoonotic impact of each species differs considerably. For example, *A*. *ceylanicum* is the only canine hookworm known to produce patent infection in humans in both natural and experimental infections and is the second most common hookworm infecting humans in the Asia Pacific [16,19,53]. Conversely, *A*. *caninum* and *A*. *braziliense* cause accidental infections in humans although preliminary evidence on the ability of the former to produce patent infections in human patients have recently been reported [23].

WHO recommends different targeted treatments in endemic populations based on the prevalence detected in people from these geographical areas [54]. However, human treatment alone is insufficient for the control of STH in the presence of zoonotic parasites such as *A*. *ceylanicum* [30,33,55]. As we approach the goal of eliminating morbidity associated with human STHs, the need to monitor both canines and humans for emerging zoonoses such as *A*. *ceylanicum* and the subsequent requirement for accurate high throughput diagnostic techniques, will become increasingly important [56].

These novel multiplex qPCRs for the four species of canine hookworms showed higher diagnostic sensitivity compared to the PCR-RFLP and have greater ability to detect mixed infections compared to previously published assays. These qPCRs will assist large epidemiological surveillance investigations, the assessment of the role of dogs in the transmission of hookworms to humans and the efficacy of anthelminthic treatments in canine populations.

## Supporting information

S1 Text Multiple alignments of the ITS-1 region of hookworm species. In grey primer pairs designed and in red probes.
(DOCX)
