# Supplementary material for: High-throughput multiplex qPCRs for the surveillance of zoonotic species of canine hookworms
Source: PLoS Negl Trop Dis. 2020 Jun 15;14(6):e0008392. doi: 10.1371/journal.pntd.0008392 (PMC7316352; doi:10.1371/journal.pntd.0008392)
Supplement: S1 Text — In grey primer pairs designed and in red probes. (DOCX) [file pntd.0008392.s001.docx]

>A_braziliense (JQ812693; DQ359149: DQ438056)

ACTTTGTCGGGAAGGTTGGGAGTATCGCCCCCGTTATAGCCCTTCTGTAAGGTGTCTATGTACAGCATGAGTCGTTCCTGGGTGGCGGCAGTGATTGCTTGTACGAAGCTCGCGGTTTCGTCAGAGCTTTAGACTTGATGAGCATTGCTAGAATGCCGCCTTACCTGCTTGTGTTGGTGGTTGAGCGCTAGGCTAACGCCTGGTGCGGCACCTGTCTGTCAGGAAACCTTAATGATCTGCTAACGCGGACGCCA

>A_caninum (KP844730; JQ812694; DQ438071)

ACTTTGTCGGGAAGGTTGGGAGTATCGCCCACCGTTACAGCCCTATGTAAGGTGTCTATGTGCAGCAAGAGTCGTTACTGGGTGGCGGCAGTGATTGCTGTGCGAAGTTCGCGTTTCGCTGAGCTTTAGACTTGATGAGCATTGCATGAATGCCGCCTTACTGCTTGTGTTGGTGGTTGAGCATTAGGCTAACGCCTGATGCGGCACCTGTCTGTCAGGAAACCTTAATGATCTGCTAACGCGGACGCCA

>A_duodenale (MK271367; EU344797)

ACTTTGTCGGGAAGGTTGGGAGTATCGCCCCCCGTTATAGCCCTACGTAAGGTGTCTATGTGCAGCAAG

AGTCGTTACTGGGTGRCGGCAGTGATTGCTGTGCGAAGTTCGCGTTTCGCTGAGCTTTAGACTTGATGAG

CATTGCATGAATGCCGCCTTACTGCTTGTGTTGGTGGTTGAGCATTAGGCTAACGCCTGATGCGGCACCT

GTCTGTCAGGAAACCTTAATGATCTGCTAACGCGGACGCCA

>U_stenocephala (AF194145; HQ262052)

ACTTTGTCGGGAAGGTTGGGAGTATCGCCCCCCTTTGAGCCCAACGTGAGGTGTCTATGTGCAGCAAGAGCCGTTTCTGGGTGGCGGCCGTGATTGCTGTGCGAAGTTCGCGTTTCGCTGAGCTTTAGACTTGATGAGCATTGCTGGAATGCCGCCTTACTGTTTGTGTTGGTGGTTGGGCATTAGGCGGCAACGTCTGGTGCGACACCTGTTTGTCAGGAAACCTTAATGATCTGCTCACGTGGACGCCA

>A_ceylanicum (DQ780009; DQ831518)

ACTTTGTCGGGAAGGTTGGGAGTATCGCCCCCCGTTACAGCCCTACGTGAGGTGTCTATGTGCAGCAAGAGCCGTTCCTGGGTGGCGGCAGTGATTGCTGTGCGAAGTTCGCGTTTCGCTGAGCTTTAGACTTGATGAGCATTGCATGAATGCCGCCTTACTGCTTGTGTTGGTGGTTGAGCATTAGGCTAACGCCTAGTGCGGCACCTGTCTGTCAGGAAACCTTAATGATCTGCTAACGCGGACGCCA

A_caninum ACTTTGTCGGGAAGGTTGGGAGTATCGCCCACCGTTACAGCCCTAT-GTAAGGTGTCTAT

A_duodenale ACTTTGTCGGGAAGGTTGGGAGTATCGCCCCCCGTTATAGCCCTAC-GTAAGGTGTCTAT

A_ceylanicum ACTTTGTCGGGAAGGTTGGGAGTATCGCCCCCCGTTACAGCCCTAC-GTGAGGTGTCTAT

U_stenocephala ACTTTGTCGGGAAGGTTGGGAGTATCGCCCCCCTTTG-AGCCCAAC-GTGAGGTGTCTAT

A_braziliense ACTTTGTCGGGAAGGTTGGGAGTATCGCCC-CCGTTATAGCCCTTCTGTAAGGTGTCTAT

****************************** ** ** ***** ** **********

A_caninum GTGCAGCAAGAGTCGTTACTGGGTGGCGGCAGTGATTGCT-GTGCGAAGTTCGCG-TTTC

A_duodenale GTGCAGCAAGAGTCGTTACTGGGTGRCGGCAGTGATTGCT-GTGCGAAGTTCGCG-TTTC

A_ceylanicum GTGCAGCAAGAGCCGTTCCTGGGTGGCGGCAGTGATTGCT-GTGCGAAGTTCGCG-TTTC

U_stenocephala GTGCAGCAAGAGCCGTTTCTGGGTGGCGGCCGTGATTGCT-GTGCGAAGTTCGCG-TTTC

A_braziliense GTACAGCATGAGTCGTTCCTGGGTGGCGGCAGTGATTGCTTGTACGAAGCTCGCGGTTTC

** ***** *** **** ******* **** ********* ** ***** ***** ****

A_caninum GCT-GAGCTTTAGACTTGATGAGCATTGCATGAATGCCGCCTTAC-TGCTTGTGTTGGTG

A_duodenale GCT-GAGCTTTAGACTTGATGAGCATTGCATGAATGCCGCCTTAC-TGCTTGTGTTGGTG

A_ceylanicum GCT-GAGCTTTAGACTTGATGAGCATTGCATGAATGCCGCCTTAC-TGCTTGTGTTGGTG

U_stenocephala GCT-GAGCTTTAGACTTGATGAGCATTGCTGGAATGCCGCCTTAC-TGTTTGTGTTGGTG

A_braziliense GTCAGAGCTTTAGACTTGATGAGCATTGCTAGAATGCCGCCTTACCTGCTTGTGTTGGTG

* ************************* ************** ** ***********

A_caninum GTTGAGCATTAGGC--TAACGCCTGATGCGGCACCTGTCTGTCAGGAAACCTTAATGATC

A_duodenale GTTGAGCATTAGGC--TAACGCCTGATGCGGCACCTGTCTGTCAGGAAACCTTAATGATC

A_ceylanicum GTTGAGCATTAGGC--TAACGCCTAGTGCGGCACCTGTCTGTCAGGAAACCTTAATGATC

U_stenocephala GTTGGGCATTAGGCGGCAACGTCTGGTGCGACACCTGTTTGTCAGGAAACCTTAATGATC

A_braziliense GTTGAGCGCTAGGC--TAACGCCTGGTGCGGCACCTGTCTGTCAGGAAACCTTAATGATC

**** ** ***** **** ** **** ******* *********************

A_caninum TGCTAACGCGGACGCCA

A_duodenale TGCTAACGCGGACGCCA

A_ceylanicum TGCTAACGCGGACGCCA

U_stenocephala TGCTCACGTGGACGCCA

A_braziliense TGCTAACGCGGACGCCA

**** *** ********
